# A Suspicious Pancreatic Mass in Chronic Pancreatitis: Pancreatic Actinomycosis

**DOI:** 10.1155/2015/767365

**Published:** 2015-02-03

**Authors:** F. de Clerck, P. Laukens, V. De Wilde, L. Vandeputte, M. Cabooter, J. Van Huysse, H. Orlent

**Affiliations:** ^1^Department of Gastroenterology and Hepatology, AZ Sint-Jan, Ruddershove 10, 8000 Bruges, Belgium; ^2^Department of Pathology, AZ Sint-Jan, Ruddershove 10, 8000 Bruges, Belgium

## Abstract

*Introduction*. Pancreatic actinomycosis is a chronic infection of the pancreas caused by the suppurative Gram-positive bacterium *Actinomyces*. It has mostly been described in patients following repeated main pancreatic duct stenting in the context of chronic pancreatitis or following pancreatic surgery. This type of pancreatitis is often erroneously interpreted as pancreatic malignancy due to the specific invasive characteristics of *Actinomyces*. *Case*. A 64-year-old male with a history of chronic pancreatitis and repeated main pancreatic duct stenting presented with weight loss, fever, night sweats, and abdominal pain. CT imaging revealed a mass in the pancreatic tail, invading the surrounding tissue and resulting in splenic vein thrombosis. Resectable pancreatic cancer was suspected, and pancreatic tail resection was performed. Postoperative findings revealed pancreatic actinomycosis instead of neoplasia. *Conclusion*. Pancreatic actinomycosis is a rare type of infectious pancreatitis that should be included in the differential diagnosis when a pancreatic mass is discovered in a patient with chronic pancreatitis and prior main pancreatic duct stenting. Our case emphasizes the importance of pursuing a histomorphological confirmation.

## 1. Introduction

Actinomycosis is a very rare, chronic, suppurative disease caused by the Gram-positive, filamentous, facultative anaerobic bacterium* Actinomyces israelii* [1].* Actinomyces* are commensals of the oropharynx, gastrointestinal and female genital tracts. Disruption of the normal mucosal integrity is a prerequisite for induction of disease. Characteristics of actinomycosis include multiple abscesses and sinus tracts that may discharge sulfur granules. Its typical granulomatous inflammation causes fibrosis and pseudotumoral mass formation.* Actinomyces* infection often mimics malignancy because of its invasive and tissue plane-disrupting properties [1–3]. Abdominopelvic actinomycosis accounts for 10–20% of reported* Actinomyces* infections. Pancreatic involvement is extremely rare and has been reported in the context of chronic pancreatitis with main pancreatic duct (MPD) stenting or prior pancreatic surgery [4].

## 2. Case Report

A 64-year-old male presented in March 2014 with nausea, weight loss, dull pain in the left upper quadrant radiating to the back, and trembling fever. His medical history consisted of diabetes mellitus, paroxysmal atrial fibrillation, and previous alcohol abuse. In 2008, he presented with repeated episodes of acute edematous pancreatitis. CT imaging revealed chronic calcifying pancreatitis with multiple stones in a dilated MPD. Extracorporeal shock wave lithotripsy in conjunction with MPD stenting was performed during repeated sessions until the MPD was cleared. This patient remained free of pancreatitis. He was lost to follow-up at the end of 2012.

**Figure 1:**
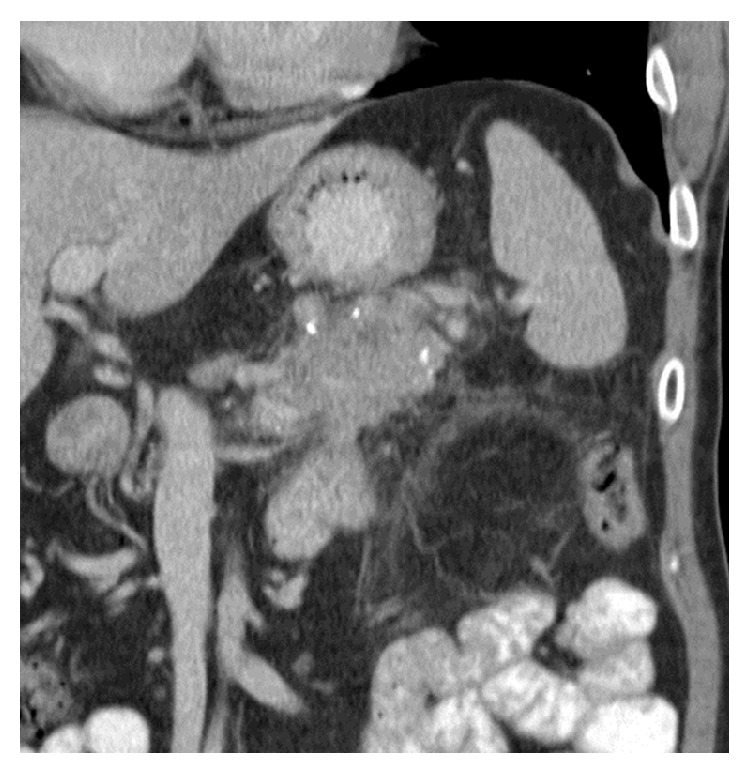
Coronal CT view: calcifying chronic pancreatitis with a heterogeneous mass in the pancreatic tail.

In March 2014, laboratory results showed an elevation of the CRP level to 180 mg/dL and a normal serum lipase value. On CT, a large heterogeneous mass in the pancreatic tail with invasion of the anterior pararenal fascia and the adjacent small bowel loop and splenic vein thrombosis were revealed (Figure 1). Both findings, which were suggestive of malignancy, were confirmed by endoscopic ultrasound (EUS). However, monolayer cytology following fine-needle aspiration (FNA) did not indicate malignancy, only showing low cellularity and large amounts of blood in the sample. The level of CA19.9 was normal and further evaluation for metastatic disease was negative. The patient was presented at our multidisciplinary pancreatic team meeting and was scheduled for pancreatic tail resection with the patient's informed consent.

**Figure 2:**
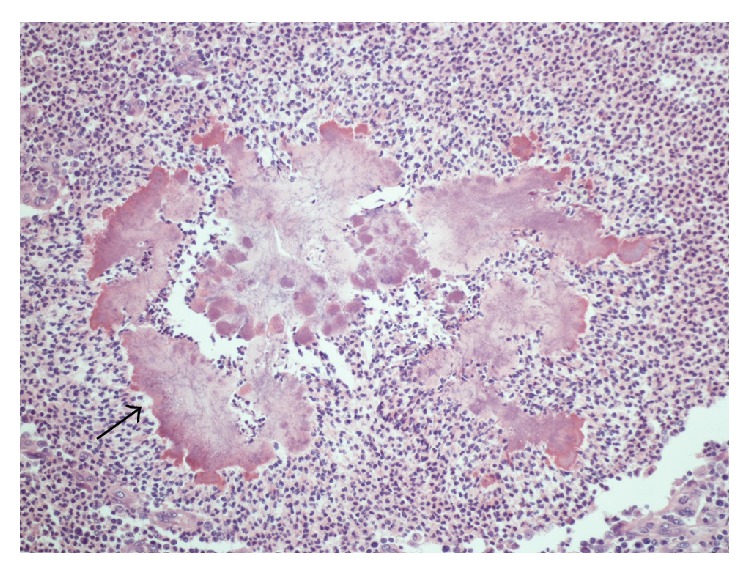
Sulfur granules consisting of a conglomeration of filamentous bacteria. The filaments are best visualized at the margins of the granules (arrow). The surrounding tissue shows dense inflammatory infiltration by lymphocytes, neutrophils, and foamy macrophages (2).

Intraoperative inspection showed edema and fibrosis of the tissue surrounding the pancreas with adherence to the stomach and small bowel. Histomorphological examination showed active granulomatous pancreatitis with small abscesses and sulfur granules (*Actinomyces* colonies) were observed, lacking evidence of malignancy (Figure 2). There was extensive fibrosis with adherence of the mass to the spleen.

The patient was started on IV amoxicillin clavulanic acid (4 × 1 gram) postoperatively, resulting in a rapid clinical response with the disappearance of all symptoms. After the initial four weeks of IV treatment, the patient was started on oral amoxicillin (3 × 1 gram) for an additional six months. He has remained asymptomatic since this time.

## 3. Discussion

Pancreatic actinomycosis is an extremely rare type of infectious pancreatitis predominantly described in the context of prior pancreas surgery or chronic pancreatitis with MPD stenting. Repeated ERCP procedures with stone extractions and MPD stenting most likely disrupted the mucosal integrity needed to initiate actinomycosis in our patient.

Symptoms of abdominal actinomycosis are nonspecific and indolent, making the diagnosis very challenging. The clinical presentation includes low-grade fever, weight loss, fatigue, a change in bowel habits, vague abdominal discomfort, nausea, vomiting, and the sensation of a mass. Infectious pancreatitis was not disregarded in our patient, the most confounding factor being the negative EUS-FNA result. Gram, Grocott's, and periodic acid-Schiff (PAS) stain did not show microorganisms. Even when* Actinomyces* would have been found in the FNA sample by stain or culture, its significance would have been unclear in the absence of sulfur granules in the FNA sample, since* Actinomyces* is a commensal of the GI tract. A second EUS-FNA was not attempted due to the presence of signs of a possible malignant process.

Long-term antibiotic treatment (6–12 months) with penicillin is the therapeutic cornerstone of the treatment of this condition [2, 3]. Surgical intervention is normally not required when the diagnosis is made preoperatively and is reserved for abscess drainage or the removal of necrotic tissue in select cases. Thus, it is important to strive for a cytologically or histologically confirmed preoperative diagnosis aimed at preventing redundant surgery, similar to the present case [5, 6]. Prognosis is excellent in cases in which the correct diagnosis and treatment are chosen.

## 4. Conclusion

Pancreatic actinomycosis should be included in the differential diagnosis when a pancreatic mass is encountered in a patient with chronic pancreatitis and a history of MPD stenting or pancreatic surgery. It is important to pursue a cytologically or histologically confirmed preoperative diagnosis because it allows for the avoidance of unwarranted surgical intervention.

## Abbreviations

MPD: Main pancreatic duct
EUS: Endoscopic ultrasound
FNA(C): Fine-needle aspiration (cytology)
ERCP: Endoscopic retrograde cholangiopancreatography.
